# Indolent Propionibacterium acnes infection associated with orthopedic hardware in the ankle: A case report and literature review

**DOI:** 10.1002/ccr3.2283

**Published:** 2019-06-24

**Authors:** Abdul R. Arain, Connor W. Sullivan, Kyle Angelicola‐Richardson, Stefanos Haddad, Andrew Rosenbaum

**Affiliations:** ^1^ Albany Medical Center Albany New York USA

**Keywords:** *P. acnes* foot and ankle infection, Propionibacterium acnes in orthopedic hardware, Propionibacterium acnes infection of the foot

## Abstract

Propionibacterium acnes (*P. acnes*) infections of the foot and ankle are very rare and require a unique approach to prevention, diagnosis, and treatment. Clinicians should consider *P. acnes* as a cause for all late and indolent orthopedic infections, as appropriate surgical and medical management can result in a good outcome.

## 1 INTRODUCTION

Postoperative infections are challenging complications following orthopedic surgery. Patients with postoperative wound infections have higher rates of long‐term wound complications, prolonged antibiotic requirements, twice the mortality rate, increased need for repeat operative procedures and are 60% more likely to be admitted to intensive care units compared to patients without postoperative infections.^1^ Identification and initiation of pathogen‐specific antibiotic treatment is of paramount importance in controlling the morbidity associated with postoperative infections.

We describe a unique case of a patient with an open ankle fracture who later developed a chronic Propionibacterium acnes (*P. acnes*) infection, necessitating a delayed ankle fusion. An indolent *P. acnes* infection was diagnosed based on cultures taken at the time of fusion, and an appropriate course of antibiotics was initiated with resolution of symptoms.

Historically, *P. acnes* has been implicated as a pathogen in brain abscesses, ocular infections, and facial acne, with bone and joint infections being less common. It is now of particular concern in shoulder arthroplasty, with the reported incidence ranging from 12% to 51.3% of prosthetic joint infections in this location.^2^, ^3^ Most patients are adequately treated with component exchange and long‐term antibiotics for 3‐6 months, with amoxicillin and rifampin being the most commonly used regimen.^4^ The pathogen more commonly colonizes the shoulder and axillary regions that have a significant amount of sebum‐rich hair follicles. Therefore, *P. acnes* infections in regions of the body relatively void of hair follicles such as the foot and ankle are rare and unexpected, posing a unique diagnostic challenge. *P. acnes* is cultured in <1.35% to 12% of total foot and ankle infections.^2^, ^5^ Furthermore, colonization may be missed because growth may take up to 15 days in enriched broth. The diagnosis may also be difficult since patients usually present with no fever or local inflammation and normal serum inflammatory markers. Recent studies involving *P. acnes* in shoulder arthroplasty infections showed that CRP and ESR were elevated in only 10% of patients.^6^ In addition to being diagnostically challenging, preventing and treating active infections are also cumbersome. Routine surface sterilization and pre‐ and postoperative antibiotics protocols are designed to target *Staphylococcus aureus* and other more common organisms encountered during foot and ankle surgery and may be insufficient at eradicating *P. acnes*.^7^, ^8^

## 2 CASE STUDY

**Figure 1:**
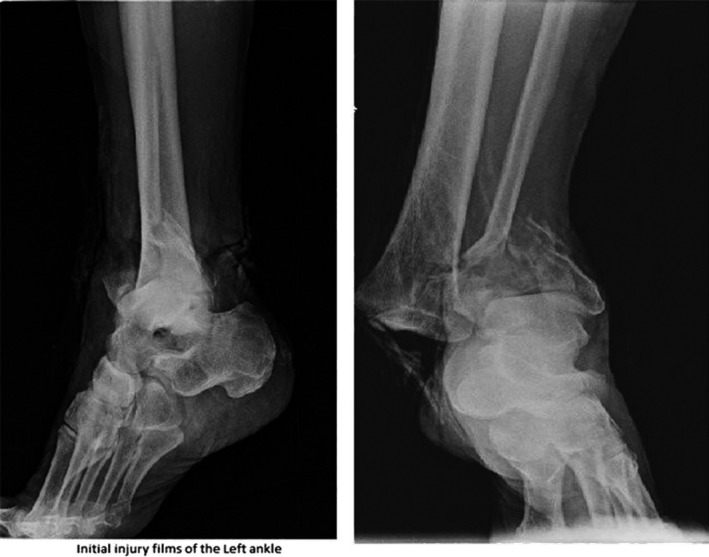
AP and Lateral injury X‐rays of the left ankle showing a ankle fracture dislocation

**Figure 2:**
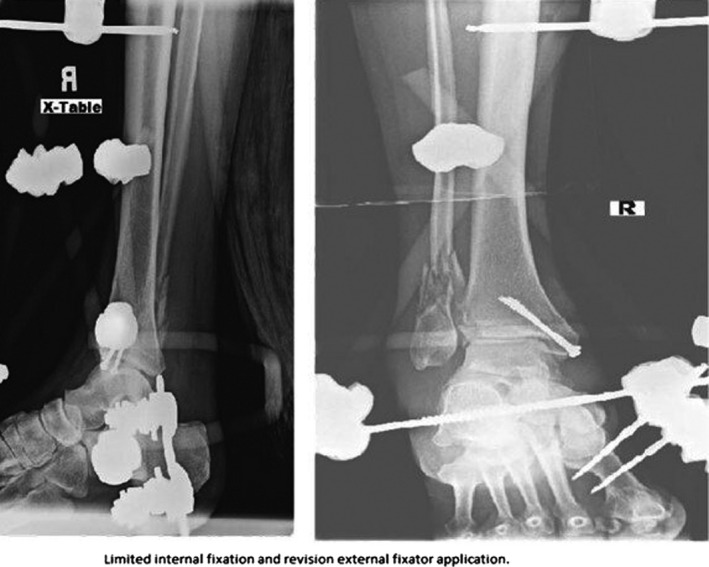
AP and Lateral X‐rays of the left ankle status postreduction of the ankle joint, placement of an external fixator and screw fixation of the medial malleolus

An 82‐year‐old male presented to our institution with an isolated open right ankle fracture dislocation seen in Figure 1, after a 5 foot fall from a ladder. There was a 5‐cm transverse laceration along the medial aspect of his ankle with exposed bone and gross contamination with dirt and debris. He was treated with intravenous Ancef prophylactically in the emergency department. He was brought to the operating room on the day of presentation for an irrigation and debridement, reduction and placement into an external fixator. The wound was closed without tension, and antibiotics were administered for 48 hours postoperatively. On postoperative day number two, there was a loss of reduction in the external fixator. The patient returned to the operating room for revision of the external fixator and irrigation and debridement. The wound bed appeared clean, and the medial malleolus was fixated using two partially threaded cannulated screws seen in Figure 2.

Twelve days after the initial presentation, the medial wound showed delayed healing, dehiscence and purulent drainage. The patient was again brought to the operating room for a thorough excisional debridement and irrigation of the wound. The wound was closed after debridement. The patient was started on broad‐spectrum antibiotics. Cultures grew Enterobacter cloacae, Enterococcus faecalis, and mucormycosis. Under the guidance of the infectious disease team, the patient was initially placed on intravenous unasyn, levaquin, and micafungin. He was then transitioned to oral augmentin, levofloxacin, and voriconazole for 6 weeks at the time of discharge.

The patient continued to show signs of healing on plain radiographs and had minimal pain. His wound was well healed with no signs of continued infection. After completion of his antibiotic course, the patient returned to the operating room for removal of the external fixator. He was allowed to weight bear as tolerated in a CAM walking boot. The patient subsequently developed valgus collapse of this distal tibia over the next 10 weeks. This was attributed to nonunion of his initial fracture. At this time, ankle fusion was considered; however, since inflammatory markers remained elevated, the patient returned to the operating room, where his medial malleolus fixation was removed, the malleolus was excised and the ankle fracture was closed reduced with placement of an external fixator to correct the valgus deformity. At this time, cultures were taken which grew *S lungdenosis* and *S capitis*. Fungal cultures were negative. He was given 6 weeks of IV antibiotic therapy at this time.

**Figure 3:**
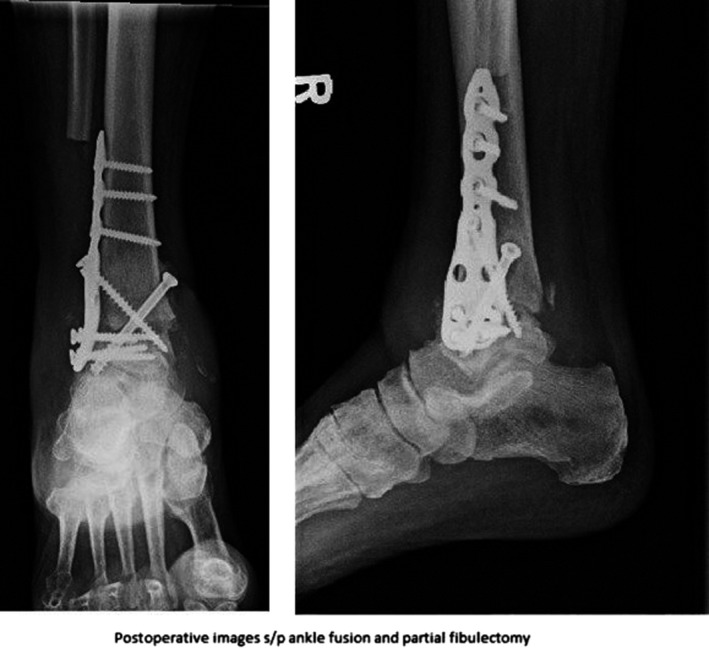
AP and Lateral X‐rays of the left ankle status postfusion of the ankle joint and partial resection of the fibula

Approximately 10 weeks later the patients’ inflammatory markers had begun to normalize. He returned to the operating room for an ankle fusion using proximal tibial autograft seen in Figure 3. Under the recommendations of infectious disease, broth from the cultures was intubated for 2 weeks and grew *P. acnes* after 12 days. The remaining cultures were negative for bacteria and fungus. He was treated with a 6‐week course of IV‐ceftin without further complication. The patient has been satisfied with the ankle fusion and has not had any recurrent infection or return to the operating room at the 1‐year follow up mark.

## 3 DISCUSSION

Propionibacterium acnes surgical site infections following foot and ankle surgery are a rare and virtually unknown entity.^2^, ^5^ More commonly the implicated pathogen in an infection after foot and ankle surgery is *S aureus*.^9^ As a result, empiric antibiotics in infectious cases are often tailored to *S aureus* and other common pathogens, leaving patients uncovered for *P. acnes*.^9^

Diagnostically, *P. acnes* poses a challenge as well. Cultures may take up to 15 days to grow *P. acnes*, necessitating laboratories to incubate samples for at least 2 weeks. Additionally, patients will rarely have elevated ESR and CRP, clouding the clinical picture.^6^ There are, however, reports of synovial IL‐6 being considerably elevated in patients with *P. acnes* infections.^10^ Therefore, clinicians should always consider *P. acnes* as a source of indolent surgical site infection following foot and ankle surgery.

Unfortunately, routine surface sterilization prior to surgery does not eradicate *P. acnes*. This is because *P. acnes* may reside within sebaceous glands and not on the epidermis. Consequently, *P. acnes* seeding occurs during an incision carried through a sebaceous gland or hair follicle.^4^, ^11^ There are reports of hydrogen peroxide surface sterilization decreasing the risk of *P. acnes* shoulder arthroplasty PJI.^12^ Perhaps, routine use of a hydrogen peroxide skin wash prior to surgery may aid in decreasing *P. acnes* infection in other anatomic locations.

In addition to posing a challenge in prevention and diagnosis, *P. acnes* infections may be difficult to treat. Common treatment has been delayed because of a delay in diagnosis.^6^ Once treatment has begun, the potential for biofilm must be addressed. In fact, *P. acnes* biofilm demonstrates substantial resistance to conventional antibiotics.^7^ Currently, clindamycin or vancomycin are first‐line treatments for deep *P. acnes* infections, although there is emerging resistance to clindamycin.^12^ There is evidence that rifampin with daptomycin can eradicate up to 67% of *P. acnes* biofilm.^8^ Clearly, there is much room for improving *P. acnes* surgical site infection strategies as no clear guidelines for treating *P. acnes* infection and biofilm exist. We recommend clinicians work closely with infectious disease specialist when *P. acnes* is encountered.

## 4 CONCLUSION

Despite accounting for a minority of cases, deep *P. acnes* infections require a unique approach to prevention, diagnosis, and treatment. Our case of an open ankle fracture complicated by a delayed *P. acnes* infection underscores the importance of considering *P. acnes* as a cause for late orthopedic infections, even in nonclassical locations such as the foot and ankle. Prompt diagnosis followed by appropriate surgical and medical management can result in a good outcome.

## CONFLICT OF INTEREST

None declared.

## AUTHOR CONTRIBUTIONS

ARA, CS, SH, SZ and AR participated in the diagnosis and treatment of this patient. All authors contributed to the preparation of this manuscript equally. ARA is the lead author and was additionally responsible for submission and making appropriate edits when needed. All authors approved the final manuscript.

## INFORMED CONSENT

Written‐informed consent was obtained from this patient authorizing the publication of case details and associated images. The study protocol was approved by the institute's committee on human research.
